# Characteristics of *Salmonella* From Chinese Native Chicken Breeds Fed on Conventional or Antibiotic-Free Diets

**DOI:** 10.3389/fvets.2021.607491

**Published:** 2021-03-23

**Authors:** Lulu Cui, Qingxiao Liu, Zhiyu Jiang, Yan Song, Shoujing Yi, Jianhua Qiu, Guijuan Hao, Shuhong Sun

**Affiliations:** Department of Preventive Veterinary Medicine, College of Animal Science and Technology, Shandong Agricultural University, Tai'an, China

**Keywords:** multilocus sequence typing, serotype, antibiotic resistance, chicken, *Salmonella*, antibiotic-free

## Abstract

*Salmonella* is a common food-borne Gram-negative pathogen with multiple serotypes. Pullorum disease, caused by *Salmonella* Pullorum, seriously threatens the poultry industry. Many previous studies were focused on the epidemiological characteristics of *Salmonella* infections in conventional antibiotic use poultry. However, little is known about *Salmonella* infections in chicken flocks fed on antibiotic-free diets. Herein, we investigated and compared *Salmonella* infections in three Chinese native breeders fed on antibiotic-free diets, including the Luhua, Langya, and Qingjiaoma chickens, and one conventional breeder, the Bairi chicken, via analyzing 360 dead embryos in 2019. The results showed that the main *Salmonella* serotypes detected in a total of 155 isolates were *S*. Pullorum (82.6%) and *S*. Enteritidis (17.4%). Coinfection with two serotypes of *Salmonella* was specifically found in Bairi chicken. The sequence type (ST) in *S*. Pullorum was ST92 (*n* = 96) and ST2151 (*n* = 32), whereas only ST11 (*n* = 27) was found in *S*. Enteritidis. The *Salmonella* isolates from three breeder flocks fed on antibiotic-free diets exhibited phenotypic heterogeneity with a great variety of drug resistance spectrum. Most of the isolates among three chicken breeds Luhua (64.9%, 50/77), Langya (60%, 12/20) and Qingjiaoma (58.3%, 7/12) fed on antibiotic-free diets were resistant to only one antibiotic (erythromycin), whereas the rate of resistance to one antibiotic in conventional Bairi chicken isolates was only 4.3% (2/46). The multidrug-resistance rate in *Salmonella* isolates from layer flocks fed on antibiotic-free diets (20.2%, 22/109) was significantly (*P* < 0.0001) lower than that from chickens fed on conventional diets (93.5%, 43/46). However, high rate of resistance to erythromycin (97.4%~100%) and streptomycin (26%~41.7%) were also found among three breeder flocks fed on antibiotic-free diets, indicating resistance to these antibiotics likely spread before antibiotic-free feeding in poultry farms. The findings of this study supplement the epidemiological data of salmonellosis and provide an example of the characteristics of *Salmonella* in the chicken flocks without direct antibiotic selective pressure.

## Introduction

*Salmonella* is a clinically common food-borne gram-negative pathogen with over 2,600 serotypes (1). It is demonstrated that *Salmonella* is predominantly found in poultry, eggs and dairy products (2). *Salmonella* species are considered as intracellular pathogens and carry a number of virulence factors for entry and survival in the intracellular environment, including *Salmonella* pathogenicity islands (SPIs) and *Salmonella* virulence-plasmids (3). *Salmonella* can spread not only horizontally but also vertically through eggs (chicken embryos) (4). When *Salmonella* colonizes the fallopian tubes, it can settle in the reproductive tract of poultry and contaminate fresh eggs, and contaminated chicken embryos may die due to the pathogenicity of *Salmonella* (5). The non-dead chicken embryos will still carry *Salmonella* after hatching, which will cause healthy chicks to be infected with *Salmonella* disease. For example, *Salmonella enterica* serovar Gallinarum biovar Pullorum (*S*. Pullorum), the causative agent of pullorum disease (PD) in chickens, results in a high mortality rate among embryos and chicks, as well as weakness and white diarrhea (6). Therefore, improper treatment of *Salmonella* infection may greatly increase cost on the disease management and flock breeding (7, 8).

However, strains of *Salmonella* spp. with antibiotic resistance are now widespread in both developed and developing countries (9). The emergence of *Salmonella* with antimicrobial resistance is mainly promoted by the use of antibiotics in animal feed to promote the growth of food animals, and in veterinary medicine to treat bacterial infections in those animals (2). This poses a high risk of zoonotic disease caused by the transmission of multidrug-resistant *Salmonella* strains from animals to humans via the ingestion of contaminated food or water (10, 11). To limit the negative impacts, the European Union Commission, U.S., China and many other countries banned antibiotics use for enhancing growth in livestock in 2006, 2017, and 2020, respectively (12–14). Recent studies have shown that antibiotic resistance patterns from agricultural settings can be indistinguishable, and a better understanding of the background data is required for effective agricultural management (15). A few studies (16, 17) investigated the characteristics and antibiotic resistance profile of *Salmonella* from antibiotic-free poultry or chicken meat. However, little is known about the characteristics of *Salmonella* in Chinese native chicken flocks reared on an antibiotic-free diet.

There are a variety of indigenous layer breeds in China, including the Luhua chicken, Langya chicken, Qingjiaoma chicken and Bairi chicken. The Luhua chicken has a unique black and white feather color and produces high-nutrition eggs. The Langya chicken has a small body size and high egg production. The Qingjiaoma chicken has cyan feet and black spots in body and feather. The Bairi chicken has a small body size and a *U*-shaped back. No antibiotics were used during the entire feeding process for the Luhua, Langya and Qingjiaoma chickens for at least 4 years. Earlier research in our previous study found that the detection rate of *Salmonella* in dead embryos could evaluate the *Salmonella* infection rate in chicken flocks (18, 19). In the current study, we mainly investigated the serotypes and antibiotic resistance profiles of *Salmonella* from dead embryos of Chinese native breeders fed on antibiotic-free or conventional diets in 2019. This study will help to supplement the epidemiological data of *Salmonella* infection in Chinese chicken flocks fed on antibiotic-free diets.

## Materials and Methods

### Samples and *Salmonella* Isolation

**Figure 1:**
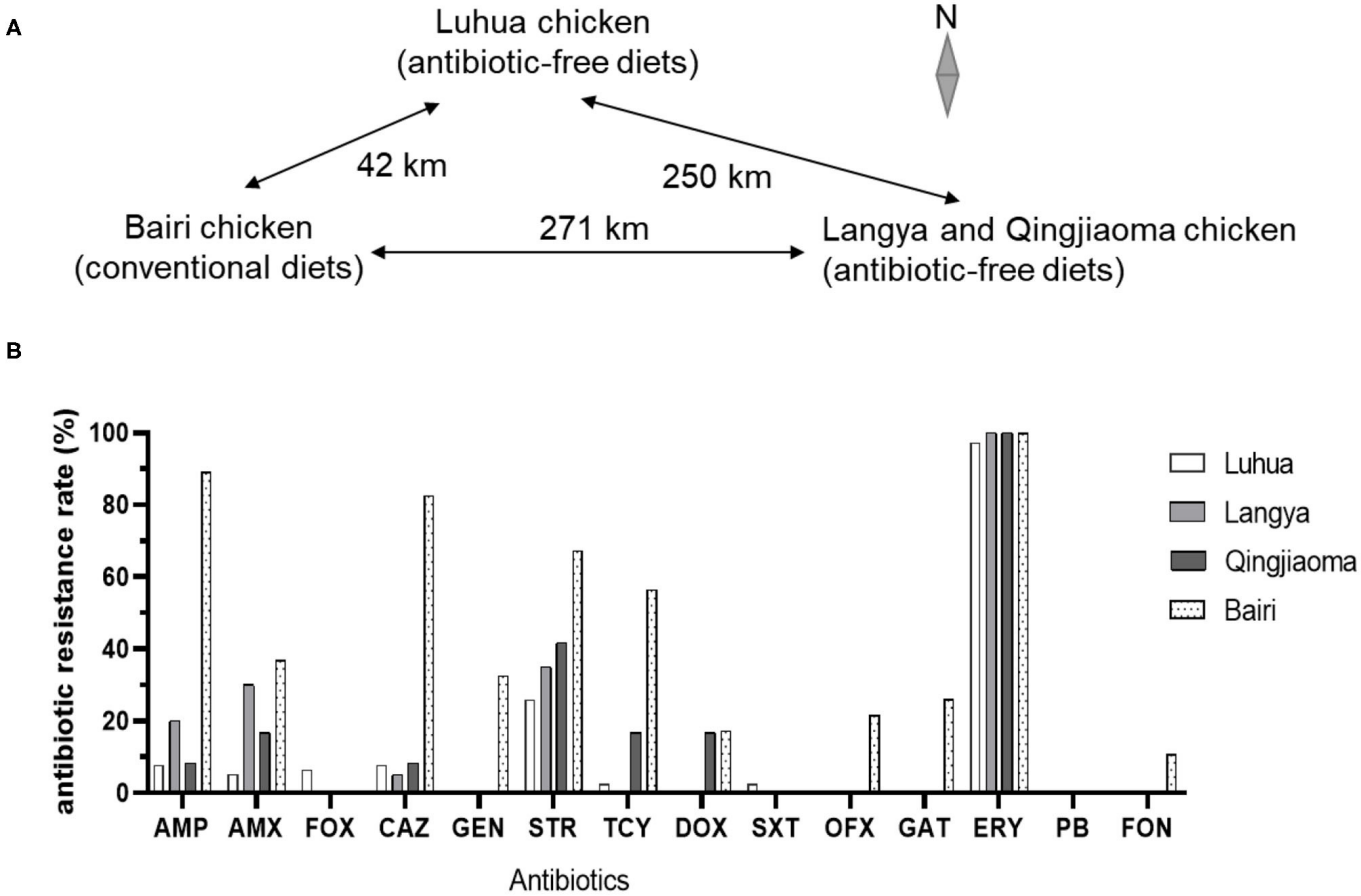
**(A)** Geographical distribution of the Chinese native layer farms in this study. **(B)** Resistance rates of *Salmonella* isolates from four Chinese native breeder flocks to different antibiotics. Antibiotic susceptibility was tested by the Kirby-Bauer disc diffusion test method and the results of antibiotic resistance rates were calculated from three repeats.

A total of 360 dead chicken embryos (18 days of incubation) were used to isolate *Salmonella* from three Chinese native layer breeders fed on antibiotic-free diets and one conventional native breeder with 90 dead embryos in each farm in 2019. In 2020, dead embryos, cloacal swabs, feed samples and waterline drip samples (nipple drinkers) were collected for *Salmonella* isolation. The Luhua breeder has been not fed with antibiotic for 6 years and its flock size is 200,000. The Langya and Qingjiaoma chickens were fed on antibiotic-free diets for 4 years in the same breeder farm with flock sizes 10,000 and 50,000, respectively. The three chicken flocks fed on antibiotic-free diets had ever used antibiotics to treat bacterial diseases before antibiotic-free feeding and they were 1-day or about 18 weeks old when the antibiotic-free diet was started. The conventional Bairi breeder farm used antibiotics to promote growth intermittently prior to this study with flock size 50,000. These native breeder flocks were generally maintained for 1.0~1.5 years, and therefore the chicken embryos from these flocks fed on antibiotic-free diets were probably in the 4th−6th generation. All of these chicken farms are located in eastern China. The Bairi chicken farm is 42 km away from Luhua layer farm and 271 km away from Langya and Qingjiaoma chicken farm (Figure 1A).

The liver, spleen and large intestine were taken from the dead chicken embryos with sterile forceps and placed in sterile microcentrifuge tubes (20). Discolored embryos, engorged blood vessels or liver necrosis were usually observed in these dead embryo samples. *Salmonella* strains were isolated from these samples using the Chinese National Standard method (GB 4789.4-2010) with some modifications. Briefly, each embryo sample was added into 4.5 mL of buffered peptone water (BPW, Land Bridge Technology, Beijing, China) and the BPW mixture was incubated at 37°C for 14 h for pre-enrichment. Approximately 0.5 mL of pre-enriched cultures were inoculated into 4.5 mL tetrathionate broth base (TTB, Qingdao Hope Bio-technology Co., Ltd.). After 20 h of incubation at 37°C for selective enrichment, one loopful of each TTB broth culture was streaked onto Xylose-Lysine-Tergitol 4 (XLT4) agar (Qingdao Hope Bio-technology Co., China) plates and incubated at 37°C for 48 h (21). About 3~5 suspected *Salmonella* colonies were identified by polymerase chain reaction (PCR) assays with primers designed for *Salmonella invA* (product of 331 bp) and *S*. Pullorum *iPAJ* (740 bp) (19). Only one colony with the same morphology per sample was picked and confirmed by MALDI Biotyper SMART (Beckman Coulter Inc, US). Bacterial DNA was extracted using TIANamp Bacterial DNA Kit (TIANGEN, Beijing, China) according to the manufacturer's instructions. PCR was performed in a 25.0 μL mixture containing 12.5 μL of 2 × Taq Master Mix (Vazyme Nanjing, China), 9.5 μL ddH_2_O, 1.0 μL of sample DNA, and 1.0 μL of each primer. PCR reactions were conducted using annealing at 55°C for *invA* and 58°C for *iPAJ*. The standard strain of *S*. Enteritidis (CVCC3377) and *S*. Pullorum (CVCC535) purchased from the China Veterinary Culture Collection Center (Beijing, China) were used as control strains.

### *Salmonella* Serotyping

According to the manufacturer's instructions from the *Salmonella* serotyping kit (Tianrun Bio-Pharmaceutical, Ningbo, China), all isolates used in this study were serotyped by slide agglutination using a commercial *Salmonella* antisera kit (Tianrun Bio-Pharmaceutical, Ningbo, China). The kit contained Vi antiserum and monovalent and polyvalent H and O antisera with a total of 60 factors. A single colony of *Salmonella* on the nutrient agar plate was mixed with polyvalent O antisera first, then with the specific monovalent antisera testing agglutination within 60 sec. Once the O and H antigens are identified, the serotype can be determined according to the Kauffmann-White scheme (22, 23).

### Multilocus Sequence Typing

Seven housekeeping genes (*aroC, dnaN, hemD, hisD, purE, sucA*, and *thrA*) were used to characterize Multilocus sequence typing (MLST) of *Salmonella* isolates according to the instructions from the University of Warwick (http://mlst.warwick.ac.uk/mlst/). The primer pairs for the PCR amplification of internal fragments of these genes were used according to the protocols on the EnteroBase website (https://enterobase.readthedocs.io/en/latest/mlst/mlst-legacy-info-senterica.html). All PCR reactions were conducted by using an annealing temperature of 55°C. Gene products were sequenced (Sangon Biotech, Shanghai, China) and the allele number of the corresponding sequence for each of the seven housekeeping genes was obtained by sequence alignment with BioEdit software based on the “*Salmonella enterica* MLST Database.” The sequence type (ST) was assigned according to the Achtman seven Gene MLST scheme as described online (http://mlst.warwick.ac.uk/mlst/dbs/Senterica) (20, 24).

### Antimicrobial Susceptibility Testing

According to the Kirby Bauer method recommended by the World Health Organization and the manual of clinical and Laboratory Standards Institute (CLSI, 2017), antimicrobial susceptibility testing of the *Salmonella* isolates obtained in this study was performed with a total of 14 antibiotics (Hangzhou Binhe Microorganism Reagant Co., Ltd., China), including ampicillin (AMP; 10 μg), cefoxitin (FOX; 30 μg), ceftazidime (CAZ; 30 μg), erythromycin (ERY; 15 μg), gentamicin (GEN; 10 μg), streptomycin (STR; 10 μg), tetracycline (TCY; 30 μg), sulfamethoxazole (SXT; 25 μg), ofloxacin (OFX; 5 μg), gatifloxacin (GAT; 5 μg), amoxicillin (AMX; 20 μg), doxycycline (DOX; 30μg), florfenicol (FON; 30 μg) and polymyxin B (PB; 300 IU) (25). *E. coli* (ATCC 25922) and (ATCC 35218) were used as quality control strains according to the CLSI M100-S27 guideline. Multiple drug resistance (MDR) was defined as bacteria isolates with resistance to one or more antibiotics in three or more antibiotic classes. The MDR rates of *Salmonella* isolates in these chicken breeds were calculated by the number of MDR isolates divided by the number of screening isolates. The total *S*. Pullorum and *S*. Enteritidis isolates from three antibiotic-free chicken breeds to different kinds of antibiotics were aggregated, respectively, and the relative antibiotic resistance rate was presented as a percentage and compared with that from the conventional Bairi chicken breed.

### Data Analysis

The Chi-squared test or Fisher's exact test were used for analyzing the data (26).

## Results

### Serotypes of *Salmonella*

In this study, a total of 155 *Salmonella* isolates were recovered from 360 dead chicken embryos from three Chinese native breeder flocks fed on antibiotic-free diets and one conventional layer breeder Bairi chicken in 2019 (Table 1 and Supplementary Table 1 in the Supplemental Material). The number of dead embryos positive for *Salmonella* was 143 (39.72%), and coinfection with two serotypes of *Salmonella* was found in 12 dead embryos (13.3%) of Bairi chicken. The 155 *Salmonella* isolates were divided into *S. Gallinarum* biovar Pullorum (*S*. Pullorum, *n* = 128) and *S*. Enteritidis (*n* = 27). The positive detection rate of *Salmonella* in the Luhua chicken (85.6%) was the highest, followed by 51.1% in the Bairi chicken, 22.2% in the Langya chicken and 13.3% in the Qingjiaoma chicken. The average isolation rate of *Salmonella* in three breeder flocks fed on antibiotic-free diets was 40.4%, lower than 51.1% in conventional Bairi chicken. *S*. Pullorum was found in all four chicken flocks, whereas *S*. Enteritidis was detected specifically in Luhua and Bairi breeder flocks. The results indicated the prevalence of *S*. Pullorum in Chinese native chicken flocks fed on both antibiotic-free and conventional diets.

**Table 1:** The serotypes, MLST and number of *Salmonella* isolates from Chinese native breeder flocks fed on antibiotic-free or conventional diets.

**Breeder**	**Antibiotic use**	**No. of isolates**			
		***Salmonella***	***S***. **Pullorum**		***S*. Enteritidis**
			**ST92**	**ST2151**	**ST11**
Luhua	No	77	71	0	6
Langya	No	20	0	20	0
Qingjiaoma	No	12	5	7	0
Bairi	Yes	46	20	5	21

In order to evaluate the *Salmonella* infection in breeder flocks after implementing the *Salmonella* eradication project and strengthening feeding management, various samples were collected from Luhua and Langya breeder flocks in 2020. Compared with the high isolation rate of *Salmonella* (85.6%) in Luhua breeder flocks in 2019, the infection rate of *Salmonella* in Luhua chicken was remarkably reduced to 2.08% (5/240) in 2020 by examining 140 dead embryos and 100 cloacal swabs (*P* < 0.0001) (Table 2). However, for Langya chicken flocks without *Salmonella* eradication project implementation, the isolation rate of *Salmonella* spp. (13.91%, 32/230) was significantly lower than that in Luhua chicken flocks (*P* < 0.0001). The 32 *Salmonella*-positive samples included 29 of 100 dead embryos (29%), 2 of 15 feed (13.33%), 1 of 100 cloacal swabs (1%) and 0 of 15 waterline drip samples (nipple drinkers) (Table 2).

**Table 2:** Comparison of the infection rates of *Salmonella* spp. from various samples in Luhua and Langya breeds in 2020.

**Breeder**	**Improvements**	**Total isolates/% (positive/total)**	**No. of isolates from various samples (positive/total)**			
			**Dead embryos**	**Cloacal swabs**	**Feed sample**	**Waterline drip**
Luhua	*Salmonella* eradication, improved feeding management	2.08% (5/240)	5/140	0/100	NA	NA
Langya	No	13.91% (32/230)	29/100	1/100	2/15	0/15

### MLST

Only three STs were identified in the 155 *Salmonella* isolates, including ST92 (61.9%, *n* = 96), ST2151 (20.6%, *n* = 32) and ST11 (17.4%, *n* = 27) (Table 1). ST92 and ST2151 were identified in *S*. Pullorum, whereas only ST11 was found in *S*. Enteritidis. For the Luhua and Bairi chickens, ST92 *S*. Pullorum was the prevalent isolate, but for the Langya and Qingjiaoma chickens, the most numerous isolate was ST2151 *S*. Pullorum.

### Antimicrobial Susceptibility Testing

**Figure 2:**
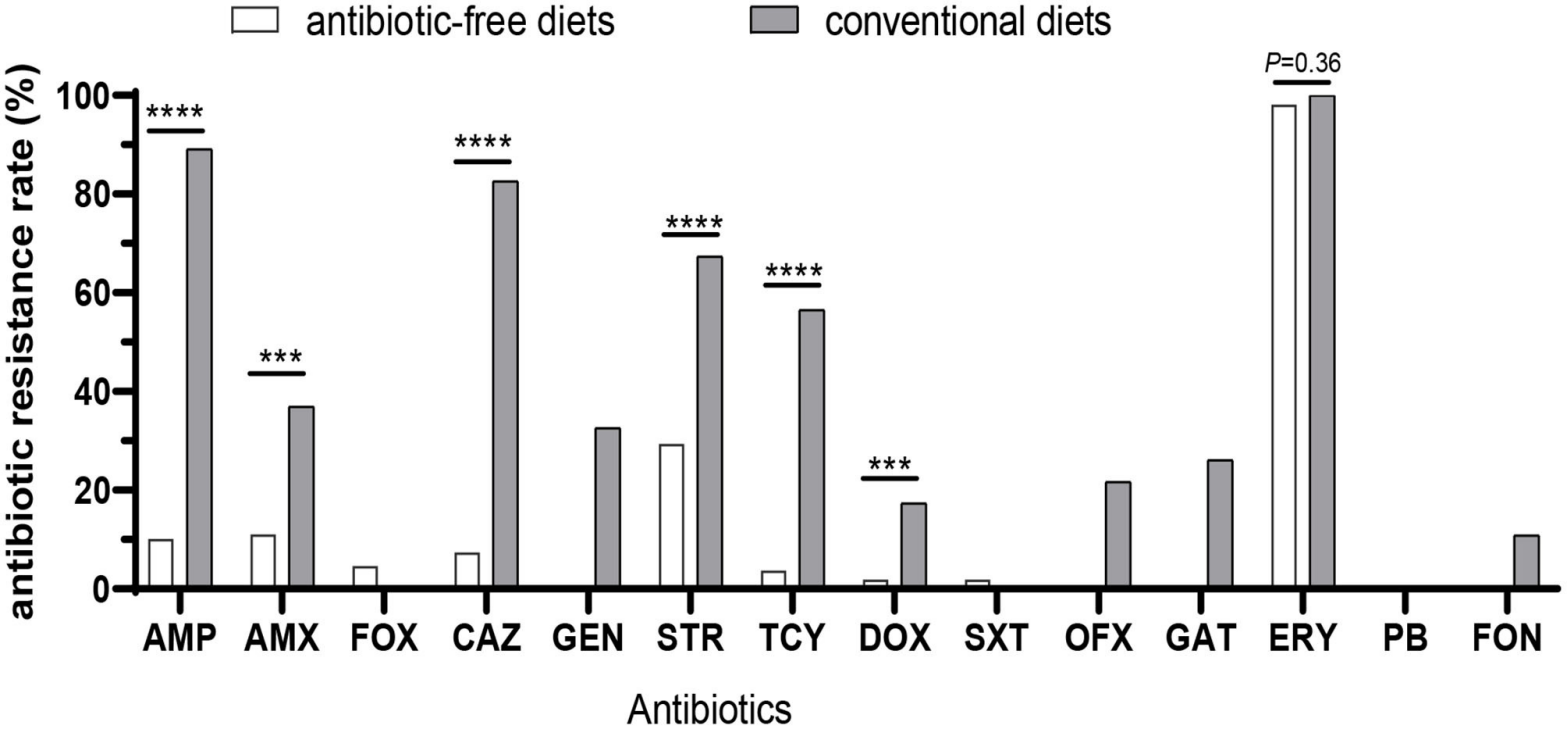
Comparison of the resistance rates of *Salmonella* isolates between three Chinese native breeder flocks fed on antibiotic-free diets and one breeder flock fed on conventional diets. The total *Salmonella* isolates from three Chinese breeder flocks fed on antibiotic-free diets were aggregated and presented as a percentage. The difference was analyzed by chi-squared test. ***P < 0.0005; ****P < 0.0001.

All of the 155 *Salmonella* isolates from chicken embryos were tested for resistance against 14 commonly used antibiotics (Figure 1B). Approximately equally high proportions of *Salmonella* isolates from three breeder flocks fed on antibiotic-free diets (98.2%, 107/109; 97.4% for Luhua, 75 of 77; 100% for Lanya, 20 of 20; 100% for Qingjiaoma, 12 of 12) and conventional Bairi chicken (100%, 46/46) were resistant to ERY (*P* = 0.36) (Figure 1B and Figure 2). A high resistance rate of *Salmonella* to STR was also found among the three breeder flocks fed on antibiotic-free diets, ranging from 26 to 41.7% (Figure 1B). But the rate of resistance to STR was significantly lower (*P* < 0.0001) in antibiotic-free-fed chicken (29.4%, 32/109) than in conventional chicken isolates (67.4%, 31/46). Moreover, the rates of resistance to AMP and AMX differed significantly (*P* < 0.0005) between chicken breeds fed on antibiotic-free diets (10.1%, 11/109; 11.0%, 12/109) and conventional Bairi chicken breed (89.1%, 41/46; 37.0%, 17/46) isolates (Figure 2), respectively. The resistant rates of *Salmonella* isolates from Bairi chicken to GEN, OFX, GAT, and FON were 10.9%~32.6%, but no isolate showed resistance to the above four antibiotics among the other three chicken breeds fed on antibiotic-free diets (Figure 1B). Among the *Salmonella* isolates from chicken breeds fed on antibiotic-free and conventional diets, the significantly different resistance rates to other antibiotics were as follows, respectively: to CAZ, 7.3% and 82.6% (*P* < 0.0001); to GEN, 0 and 32.6%; to TCY, 3.7% and 56.5% (*P* < 0.0001); to DOX, 1.8% and 17.4%; to OFX, 0 and 21.7% and to GAT, 0 and 26.1% (Figure 2). All *Salmonella* isolates used in this study were sensitive to PB, which was a drug of last resort for the treatment of MDR *Enterobacteriaceae* infection. Moreover, the rates of resistance in antibiotic-free-fed chicken and conventional chicken isolates to FOX (4.6% and 0, respectively) and SXT (1.8%, 0, respectively) were low (Figure 2).

**Figure 3:**
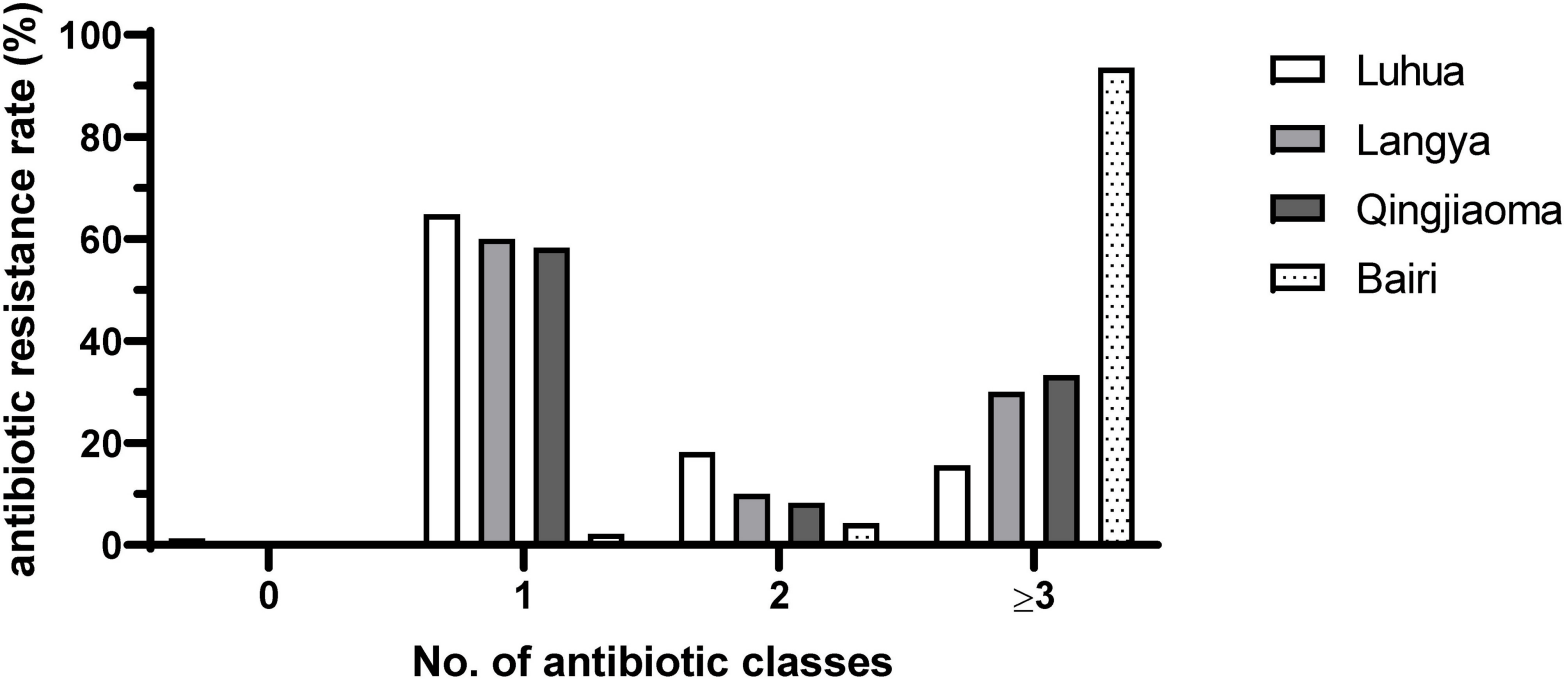
Summary of resistance rates of *Salmonella* isolates to one, two and three or more antimicrobial classes from four Chinese native breeder flocks in this study. The percentages of resistance to one, two and three or more antimicrobial classes were analyzed by the number of isolates divided by the number of all screening isolates from the chicken flocks.

**Figure 4:**
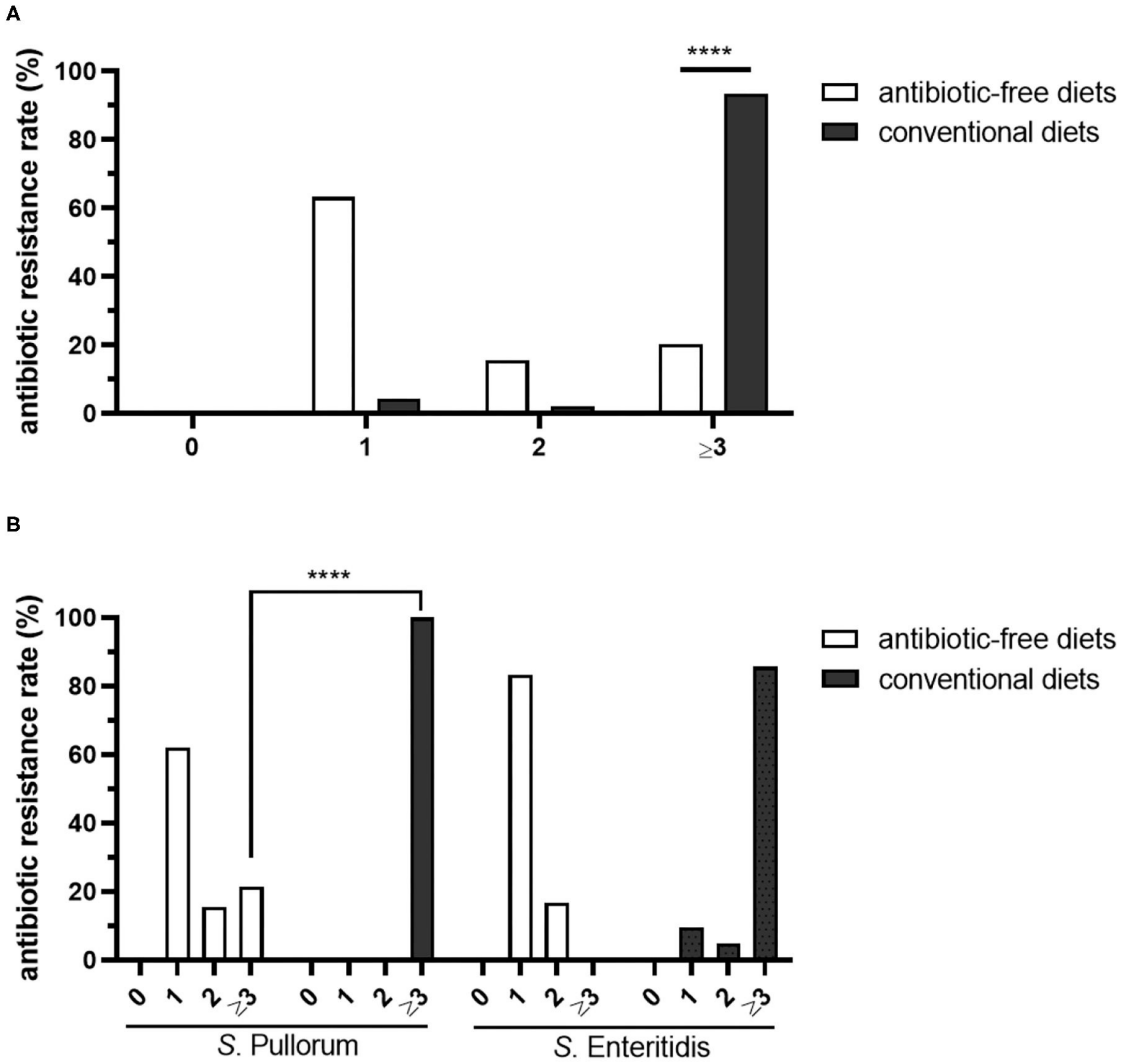
Statistics of antibiotic resistance rates of *Salmonella* isolates from Chinese native breeder flocks fed on antibiotic-free diets or conventional diets. **(A)** Comparison of the antibiotic resistance rates of *Salmonella* isolates. **(B)** Antibiotic resistance rates of different *Salmonella* serotypes. The total *Salmonella* spp., *S*. Pullorum and *S*. Enteritidis isolates from three antibiotic-free-fed breeder flocks to different kinds of antibiotics were aggregated together respectively, and the difference of resistant rates to antibiotics in isolates from breeder flocks fed on antibiotic-free or conventional diets was analyzed with chi-squared test. ****P < 0.0001.

Most of the isolates among three chicken breeds Luhua (64.9%, 50/77), Langya (60%, 12/20) and Qingjiaoma (58.3%, 7/12) fed on antibiotic-free diets were resistant to only one antibiotic (ERY), whereas the rate of resistance to one antibiotic in conventional Bairi chicken isolates was only 4.3% (2/46) (Figure 3). The largest proportion in conventional Bairi chicken was occupied by MDR isolates, up to 93.5% (43/46). However, the MDR rate in Luhua, Langya and Qingjiaoma chicken isolates was 15.6% (12/77), 30% (6/20) and 33.3% (4/12), respectively (Figure 3). The total MDR rate in isolates from chickens fed on antibiotic-free diets (20.2%, 22/109) was significantly (*P* < 0.0001) lower than that from chickens fed on conventional diets (93.5%, 43/46) (Figure 4A). One isolate (1/77) from the Luhua chicken was shown to be susceptible to all antibiotics tested in this study (Figure 3). Moreover, the MDR profile of both *S*. Pullorum and *S*. Enteritidis isolated from chickens fed on antibiotic-free diets exhibited a diverse drug resistance spectrum (Supplementary Table 1). By comparing the *S*. Pullorum isolates from chicken flocks fed on conventional and antibiotic-free diets, we showed that the MDR rate of conventional breeder chicken isolates (100%, 25/25) was much higher than that from three breeder flocks fed on antibiotic-free diets (21.4%, 22/103) (*P* < 0.0001) (Figure 4B). Approximately 62.1% of *S*. Pullorum isolates from three breeder flocks fed on antibiotic-free diets were resistant to only one antibiotic ERY, followed by 15.5% of *S*. Pullorum isolates that were resistant to two antibiotics tested in this study (Figure 4B). For *S*. Enteritidis, the MDR rate from the conventional Bairi chicken was up to 85.7% (18/21), whereas no isolate with MDR was found among a total of 6 isolates from three chicken breeds fed on antibiotic-free diets (Figure 4B).

## Discussion

Among three chicken breeds (Luhua, Langya and Qingjiaoma) fed on antibiotic-free diets and one conventional Bairi chicken, different serotypes and ST types of *Salmonella* were identified. However, the dominating serotype among these breeder flocks in this study was *S*. Pullorum, which was significantly different from the prevalent isolates (100% *S*. Enteritidis) from fecal swabs and chicken embryos of large-scale breeder farms in China (19). Zhao et al. (27) investigated the prevalence and characteristics of *Salmonella* in free-range chickens in China and showed that a total of 38 *Salmonella* isolates (38/300, 12.7%) were recovered and the most common serotype was *S*. Enteritidis (81.6%). Certainly, some *Salmonella* species may be missed in these flocks due to the choice of methods and media used for isolation of *Salmonella*. We were not able to differentiate the susceptibility of these chicken breeds to *Salmonella* infection due to lack of data in the study. However, the *Salmonella* infection rates in embryos were higher than that in samples from cloacal swabs and farm environments. These farms were located in different regions of China east, and different native breeder flocks had different diets with or without antibiotics added, so it seemed that the infection rate of *Salmonella* may be significantly associated with geographical distribution and feeding management level in China (18). The prevalence of *Salmonella* associated with chick mortality at hatching was investigated in three hatcheries in Jos, central Nigeria. The results showed that 45(9%) of the 500 samples were positive for *Salmonella* and the prevalent serotypes were *S*. Kentucky (75.6%) and *S*. Hadar (24.4%) (28). Bailey et al. (29) tracked the serotype of *Salmonella* through integrated broiler chicken operations in the US. The results showed that the rate of *Salmonella*-positive samples from the hatchery in 1999–2000 was the highest and the predominant serotype found in hatchery samples was *S*. Senftenberg. An association between the serotypes found in the hatchery and those found on the final processed carcasses was observed.

PD caused by *S*. Pullorum is strongly associated with vertical transmission directly from contamination of the egg in the genital tract or indirectly from chick-to-chick contact in the hatchery (30). It was demonstrated that *S*. Pullorum colonized both the ovary and the oviduct of hens and led to 6% of laid eggs being infected by *S*. Pullorum via more than one mechanism of egg infection (31). *S*. Pullorum is not excreted extensively in the feces, unlike many other *Salmonella* serotypes that are more frequently associated with human food poisoning (32). In the current study, *S*. Pullorum isolates from dead embryos in these chicken flocks exhibited limited genetic diversity in ST and only ST92 and ST2151 were determined among the total 128 isolates. Hu conducted the whole-genome sequencing of a panel of 97 *S*. Pullorum isolates between 1962 and 2014 from four countries across three continents, and Hu also found most of the strains belonged to ST92 (33). However, the *S*. Pullorum isolates in these chicken flocks fed on antibiotic-free diets exhibit phenotypic heterogeneity with relatively low antibiotic resistance rates, providing an example of *Salmonella* characteristics for the chicken production system without direct selective pressure. The most effective means of controlling pullorum disease is a combination of stringent management procedures and eradication by a serological test (34). In the United States, PD was brought under control after the implementation of the National Poultry Improvement Plan and the vaccination of flocks (35). The European Union also established a regulation focused on preventing, monitoring or eradicating *Salmonella* in poultry, and the incidence of salmonellosis had decreased since 2003 (36). However, related control programs for *S*. Pullorum eradication and available vaccines were still absent in China. The positive rate of *Salmonella* isolates from Luhua chicken in 2019 was the highest, however, *Salmonella* serological tests had been regularly done to eliminate positively infected chickens promptly since 2019. And with daily feeding management strengthening, the infection rate of *Salmonella* in Luhua chicken was dramatically reduced to 2.08% in the survey of 2020, lower than 13.91% in Langya chicken.

*S*. Enteritidis is the serovar most frequently associated with egg infection due to its unique long term ability to colonize the ovary and the oviduct of laying hens and its spread and persistence in the parental breeder flock population (37). The frequency of egg contamination by *S*. Enteritidis depends on the level of contamination of the flock, and eggs are more likely to become internally contaminated around the onset of lay (38, 39). The isolation rate of *S*. Enteritidis in the present study was 17.4% (27/155) and all were resistant to erythromycin. This was quite different from a survey with rectal swabs collected from three Chinese large-scale conventional chicken farms, which showed 80.8% (63/78) of MDR isolates with the most common serovar being *S*. Enteritidis (88.5%) (21). Unlike *S*. paratyphoid serovars that only infect humans by causing enteric fever, *S*. Enteritidis is a zoonotic pathogen of substantial concern to global human and animal health (40). Many studies using whole genome sequencing linking epidemiology, phylogeny and virulotyping are performed with *Salmonella* isolates from the harmonized monitoring of poultry and from human disease to facilitate attribution studies and identify trends associated with virulence and stress-response genes (41, 42).

Among the 14 antibiotics used in this study, the resistance rates of all *Salmonella* isolates to 11 antibiotics in conventional chicken were higher than those from chickens fed on antibiotic-free diets. The average MDR rate (20.2%, 22/109) of *Salmonella* isolates from chickens fed on antibiotic-free diets was significantly lower than the rates of 100% among 63 isolates examined by Yang et al. (19) from conventional farms in a similar geographical region, and also lower than that from other poultry farms in China (43). These data indicated that the use of antibiotics may promote the development of MDR *Salmonella* (44). Liu et al. (45) found high abundances of aminoglycoside, sulfonamide and tetracycline resistance genes in one antibiotic-free layer farm without direct antibiotic selective pressure. Similarly, the high resistance rate to streptomycin belonging to aminoglycoside antibiotic was also found in *Salmonella* isolates from antibiotic-free-fed layer farms in this study. Besides, high resistance rate to erythromycin was seen amongst three antibiotic-free-fed layer flocks in this study. The mechanisms of erythromycin resistance in *Salmonella* contain modification of the ribosomal target of macrolides and hydrolysis of the macrolide lacton ring catalyzed by erythromycin esterases (such as *ereA* and *ereB*) (46). Modification of the ribosomal target of macrolides is a common mechanism, and confers broad cross-resistance to macrolide-lincosamide-streptogramin antibiotics. This modification can occur by mutation and methylase encoded by *erm* (erythromycin ribosome methylase) genes (47). Antibiotics were used to treat bacteria diseases in the three flocks of layer breeder in this study 4–6 years ago, but those resistance antibacterial and/or genes may still circulate within the living environment of flocks. Management practices and contaminated eggs, and feces or wastewater have been attributed to the spread and persistence of antibiotic resistant *Salmonella* in the environment (2, 44). Together, these data provided an example of the *Salmonella* antibiotic resistance profiles in the chicken flocks fed on antibiotic-free diets. To the best of our knowledge, there have been no previous studies that investigated the characteristics of *Salmonella* infection in such native layer flocks fed on antibiotic-free diets in China.

In summary, the current study showed that majority of *Salmonella* isolates from three Chinese native breeder flocks fed on antibiotic-free diets were ST92 and ST2151 *S*. Pullorum and ST11 *S*. Enteritidis. The antibiotic resistance rates and MDR rates in three chicken breeds fed on antibiotic-free diets were significantly lower than that from a conventional Bairi chicken farm. Moreover, the *Salmonella* isolates in these chicken flocks fed on antibiotic-free diets exhibit phenotypic heterogeneity with a diverse drug resistance spectrum, providing an example for the occurrence of antibiotic resistance in the chicken production system without direct selective pressure.

## Data Availability Statement

The original contributions generated for this study are included in the article/Supplementary Material, further inquiries can be directed to the corresponding author/s.

## Ethics Statement

The animal study was reviewed and approved by Animal Care and Use of Shandong Agricultural University (SDAUA-2018-027). Written informed consent was obtained from the owners for the participation of their animals in this study.

## Author Contributions

LC and QL performed the experiments and analyzed the data. LC and GH worked on the manuscript writing. SS designed the experiments and worked on the manuscript. ZJ, YS, and SY helped do the experiments. JQ provided support for experiments. All authors read and approved the final manuscript.

## Conflict of Interest

The authors declare that the research was conducted in the absence of any commercial or financial relationships that could be construed as a potential conflict of interest.
